# A Multiple-SNP Approach for Genome-Wide Association Study of Milk Production Traits in Chinese Holstein Cattle

**DOI:** 10.1371/journal.pone.0099544

**Published:** 2014-08-22

**Authors:** Ming Fang, Weixuan Fu, Dan Jiang, Qin Zhang, Dongxiao Sun, Xiangdong Ding, Jianfeng Liu

**Affiliations:** 1 Key Laboratory of Animal Genetics and Breeding of Ministry of Agriculture, National Engineering Laboratory for Animal Breeding, College of Animal Science and Technology, China Agricultural University, Beijing, China; 2 Life Science College, Heilongjiang Bayi Agricultural University, Daqing, China; University of Queensland, Australia

## Abstract

The multiple-SNP analysis has been studied by many researchers, in which the effects of multiple SNPs are simultaneously estimated and tested in a multiple linear regression. The multiple-SNP association analysis usually has higher power and lower false-positive rate for detecting causative SNP(s) than single marker analysis (SMA). Several methods have been proposed to simultaneously estimate and test multiple SNP effects. In this research, a fast method called MEML (Mixed model based Expectation-Maximization Lasso algorithm) was developed for simultaneously estimate of multiple SNP effects. An improved Lasso prior was assigned to SNP effects which were estimated by searching the maximum joint posterior mode. The residual polygenic effect was included in the model to absorb many tiny SNP effects, which is treated as missing data in our EM algorithm. A series of simulation experiments were conducted to validate the proposed method, and the results showed that compared with SMMA, the new method can dramatically decrease the false-positive rate. The new method was also applied to the 50k SNP-panel dataset for genome-wide association study of milk production traits in Chinese Holstein cattle. Totally, 39 significant SNPs and their nearby 25 genes were found. The number of significant SNPs is remarkably fewer than that by SMMA which found 105 significant SNPs. Among 39 significant SNPs, 8 were also found by SMMA and several well-known QTLs or genes were confirmed again; furthermore, we also got some positional candidate gene with potential function of effecting milk production traits. These novel findings in our research should be valuable for further investigation.

## Introduction

Single marker analysis (SMA) is the most practical way for genome-wide association study (GWAS), in which each SNP is tested at a time along the genome. Assuming SNP is in linkage disequilibrium with a casual mutation or just a casual mutation, the association of a functional gene can be tested with its nearby SNP. Although the SMA provides a simple and fast way for genome-wide QTL mapping, it neglects the effects of other genes on the genome when only one SNP is tested. Because a single SNP only explains a very small portion of genetic variation, SMA may not be powerful for identifying weaker associations that may result from small allelic effects, low minor allele frequencies (MAF), or weak correlations with genotyped markers [1].

Compared with SMA, the multiple-SNP association study can simultaneously consider multiple QTL effects, and thus can reduce the estimate of error variance, and in turn increases the power to detect weaker associations and decrease the false-positive rate for QTL detection [1]. Many researchers have investigated the multiple-SNP association method [2]. These methods included multiple SNP effects in a linear model and used special model selection or shrinkage estimate methods for estimating multiple SNP effects simultaneously. Logsdon et al. [3] adapted the Bayesian classification model of Zhang et al. [4] to a (VB) for a genome-wide association study of human data. VB estimates the posterior expectation by iterative calculation and avoid the time-consuming Markov chain Monte Carlo (MCMC) algorithm, and thus, it is suitable for large number of SNPs. Wu et al. used Lasso penalized logistic regression for genome-wide association study of multiple main-effect and interacting-effect SNPs in case-control design [5]. A Bayesian Lasso technology was used by Li et al. for shrinkage estimate of multiple-SNP effects for human body mass index [6]. Before the Lasso estimation, a preconditional procedure is preformed via a supervised principal component analysis to reduce the effect of observational noise on model selection, which could denonise the response variable so that variable selection became more efficient.

The genome-wide association study of economic important traits of domestic animals has been conducted by many researches [7]. As we know, the variance of a quantitative trait locus (QTL) for a complex trait is usually small, which may increase the difficulties for detecting causative SNPs; furthermore, the traditional SMA usually generates many false-positive signals. Thus, it is meaningful to apply the multiple-SNP association method for genome-wide association study in domestic animals.

In this research, we propose an Expectation-Maximization algorithm [8] called MEML for simultaneously shrinkage estimate of multiple marker effects, which employs an improved Lasso prior for marker effect [9]. To account for many tiny-effect SNPs, the random polygenic effect is also considered in model. A series of simulation experiments are conducted to compare the proposed method and single marker mixed model method (SMMA). We also apply the proposed method to a real SNP-panel dataset from Chinese Holstein dairy cattle, in which, five milk production traits including milk yield, protein yield, fat yield, protein percentage and fat percentage are measured [7]. To obtain the significant threshold value, one thousand permutations are performed.

## Results

### The QTL-MAS XII workshop data

The simulated dataset includes six chromosomes with a total length of 600 cM, on which.6,000 SNP markers are distributed with average marker interval of 0.1 cM. The pedigree spans four generations and includes 4,665 individuals with known genotypes and phenotypic values. The detailed descriptions of the dataset can be found from its official website (http://www.computationalgenetics.se/QTLMAS08/QTLMAS/DATA.html) [10]. Although our method can simultaneously estimate all marker effects, it is too time consuming to obtain the significant threshold value by permutation test. Hence, we used a two-stage procedure for multiple-SNP modeling. We first selected 500 SNPs with the lowest *p* values in single-marker mixed-model analysis (SMMA), and then simultaneously analyzed these SNPs using our multiple-SNP method. It is a similar strategy adapted by [6].

**Figure 1:**
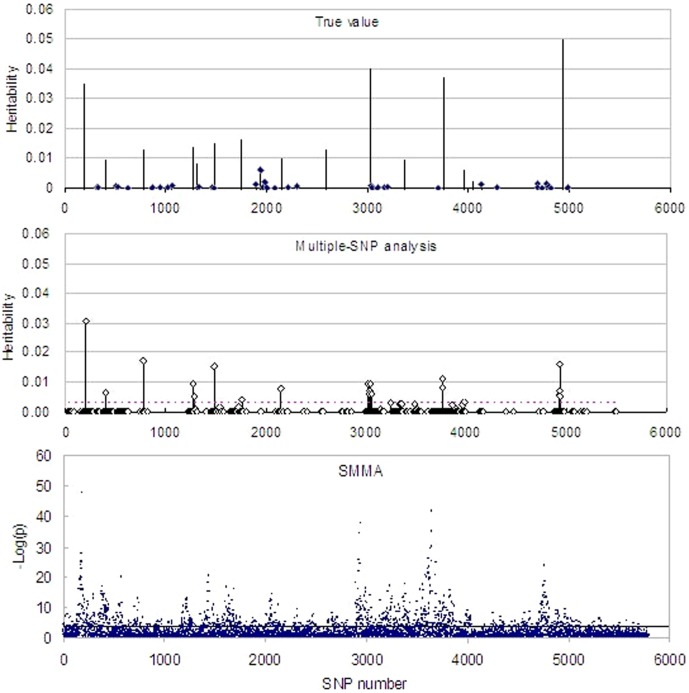
The profiles of the true SNP parameters (the top panel), the estimated 500 SNP heritabilities with MEML (the middle panel) and −log_10_^P^ with SMMA (the bottom panel), respectively. The x-axis indicates the SNP numbers. In the top panel, the true heritabilities of small-effect SNPs are presented with diamonds on the top of their needles but not for large-effect SNPs. The dotted horizontal lines in the middle and the bottom panels present the thresholds with 1,000 permutations from the multiple-SNP and SMMA methods, respectively.

Figure 1a and b show the profiles of the true heritabilities and the estimated heritabilities for the 500 SNPs with lowest *P*-values in SMMA. Many SNP heritabilities (effects) were shrunk to zero except several large-effect SNPs. Eleven SNPs were found to be significant out of the 15 simulated major-effect QTLs, and except several SNPs closely linked with the major-effect QTLs only 3 false -positive signals are showed.

Figure 1c shows the profile of −log_10_ *p* values from SMMA, where the threshold was determined with 1,000 permutations. It can be seen that almost all the simulated QTL were detected, but hundreds of false-positive signals were shown.

### Replicated simulation experiments

To further validate the efficiency of the proposed method, we employed replicated simulation experiments to test the true-positive rate (power) and the false-positive rate. A population included two generations each had 2,000 individuals were simulated. The first generation included 50 full-sib populations each with 40 individuals; and the second generation included 20 full-sib populations each with 100 individuals. The genome and QTL (position and effect) were simulated according to QTL-MAS XII dataset. We totally simulated 6 chromosomes each contained 1,000 evenly spaced SNP markers with total length of 1M. The markers in disequilibrium were created using the gene-dropping method [11]. Forty-tree markers closed to the QTL position in QTLMAS dataset were chosen to serve as QTL; the QTL effects were the same as those in QTLMAS dataset. The population mean was set as 1; and the residual error followed normal distribution with mean zero and variance 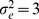. The phenotypic value for each individual was simulated by summing the population mean, QTL effects and residual error. One hundred replicated simulation experiments were conducted to compare the power and false positive between SMMA and MEML. A marker at the true QTL positions or nearby the true QTL positions with ±1 locus was defined as QTL locus; and other markers were treated as non-QTL locus. The total power for all QTL was 24.8%, which was summarized with the total number of positive QTL (1,068) divided by the total number of simulated QTL for 100 replications (43*100); and the false positive rate was 0.011%, which was summarized with the total number of false-positive QTL (63) divided by the total number of non-QTL locus, (6,000-43*3)*100, in 100 replications with MEML. Using the same way, the power and the false positive rate of SMMA were summarized and they were 35.9% (1,544 significant SNP) and 2.66% (15,638 false positive SNPs), respectively. It is noted that the power of both methods were low, since only 15 out of 43 simulated QTL had major effect. The results showed that SMMA had higher power than MEML; however, the false positive number of SMMA was much higher than MEML. It seemed that our method can provide a more conservative way for QTL detection.

### Chinese Holstein cattle data

The Chinese Holstein cattle population contained 14 sires and their 2,093 daughters, and the numbers of daughters of the 14 sires ranged from 83 to 358. The estimated breeding values (EBVs) of five milk production traits, including milk yield (MY), fat yield (FY), protein yield (PY), fat percentage (FP), and protein percentage (PP) were used as phenotypes in this study Each individual was genotyped for 54,001 SNP markers using the Illumina BovineSNP50 BeadChip (see [7] for detailed data description). The quality control procedure excluded individuals with >10% missing genotypes and SNPs with (1) call rate less than 90%, or (2) the minor allele frequency (MAF) less than 3%, or (3) the *p*-value of the Hardy-Weinberg Equilibrium (HWE) test less than 10^−6^. Eventually, 40,829 SNPs remained for the subsequent analyses. We selected 500 SNPs with the lowest *p*-values in SMMA, and then simultaneously analyzed them using the proposed method.

**Figure 2:**
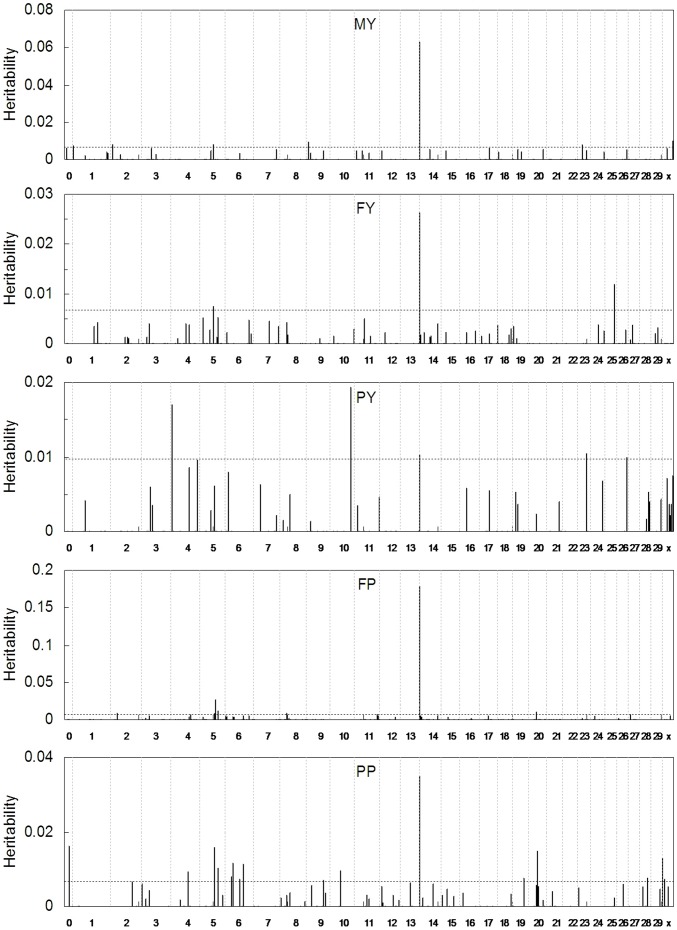
The profiles of the estimated heritabilities of 500 SNPs for five milk production traits against on the selected SNPs. The panels from the top to the bottom are the estimated heritiabilities for milk yield, fat yield, protein yield, fat percentage and protein percentage traits, respectively. The x-axis indicates the chromosome number (chromosome are divided by vertical dotted lines). The dotted horizontal line presents the threshold from 1,000 permutations.

Figure 2 shows the estimated heritabilities for the 500 SNP passing the first filter of SMMA with MEML. The threshold values for MY, FY, PY, FP and PP were 6.77×10^−3^, 6.77×10^−3^, 9.67×10^−3^, 6.67×10^−3^ and 6.66×10^−3^, respectively. Compared with the results of SMMA which found dozens of significant SNPs in some small regions,,only 1, 2 or several representative SNPs which has high possibility to harbor functional mutation(s) in these regions were found with MEML. In total, 39 SNPs were detected to be significantly associated with milk production traits; however, overall 105 significant SNPs were found with SMMA [7]. These results also reflected that our method was more conservative than SMMA. This conclusion was consisted with the previous simulation studies.

Table 1 shows the significant SNPs and 8 of them were also detected with SMMA. The Biomar website (http://www.biomart.org/) was used to search the SNP nearby (±200 kb) these genes. The SNP ARS-BFGL-NGS-4939 which is located within *DGAT1* (diacylglycerol O-acyltransferase 1) were found to have very large effects on all five milk production traits. The function of other genes associated each milk yield trait will be discussed later.

**Table 1:** The significant SNPs and their nearby genes in the analysis of Chinese dairy cattle data.

Trait	SNP	Chr	Position (bp)	Heritability	Nearest Gene	
					Gene	Distance(bp)
MY	ARS-BFGL-NGS-4939^a^	14	1801116	6.26E-02	*DGAT1*	Within
	Hapmap47777-BTA-91000	X	1.41E+08	9.81E-03	*LOC785455*	81004
	ARS-BFGL-NGS-49079^a^	9	6574398	9.05E-03	NA	NA
	ARS-BFGL-NGS-103091	5	74518588	8.14E-03	*RBFOX2*	45586
	Hapmap60955-rs29022431	23	21292766	7.83E-03	NA	NA
	ARS-BFGL-NGS-11319	2	6763227	7.69E-03	*SLC40A1*	22898
	Hapmap48369-BTA-50306	1	7627111	7.39E-03	NA	NA
FY	ARS-BFGL-NGS-4939^a^	14	1801116	2.63E-02	*DGAT1*	Within
	Hapmap42263-BTA-60093	25	35342491	1.18E-02	*MIR2388*	42285
	Hapmap40191-BTA-73919^a^	5	71978791	7.54E-03	*SYN3*	52073
PY	ARS-BFGL-BAC-6525	10	92127288	1.94E-02	*NRXN3*	Within
	ARS-BFGL-NGS-115291	4	4090824	1.70E-02	NA	NA
	ARS-BFGL-NGS-39539	23	41457147	1.05E-02	*JARID2*	154416
	ARS-BFGL-NGS-4939^a^	14	1801116	1.02E-02	*DGAT1*	Within
	ARS-BFGL-NGS-110497	26	45870133	9.98E-03	*ADAM12*	Within
	ARS-BFGL-NGS-29581	4	1.14E+08	9.66E-03	*KCNH2*	Within
FP	ARS-BFGL-NGS-4939^a^	14	1801116	0.179094	*DGAT1*	Within
	Hapmap50271-BTA-17442	5	81903458	2.68E-02	*CCDC91*	Within
	ARS-BFGL-NGS-111443	5	94269370	1.21E-02	*DERA*	46923
	Hapmap51303-BTA-74377^a^	5	83790390	1.18E-02	*ITPR2*	Within
	ARS-BFGL-NGS-118998	20	32030332	1.03E-02	*GHR*	Within
	Hapmap39717-BTA-112973	2	26781358	8.62E-03	*KBTBD10*	Within
	BTB-00231742	5	77095345	8.15E-03	NA	NA
	BTB-00285653	8	30036807	7.72E-03	*NFIB*	Within
	BTB-00777571	20	34017024	6.96E-03	NA	NA
	ARS-BFGL-NGS-113507	11	98407974	6.38E-03	*PTRH1*	Within
PP	ARS-BFGL-NGS-4939^a^	14	1801116	3.52E-02	*DGAT1*	Within
	BTA-39609-no-rs	0	0	1.63E-02	*—*	*—*
	Hapmap48524-BTA-92140^a^	5	75684520	1.58E-02	*NCF4*	24751
	BTA-50402-no-rs^a^	20	34451383	1.50E-02	NA	NA
	BTB-01844123	X	307557	1.30E-02	NA	NA
	BTA-121739-no-rs^a^	6	38063313	1.17E-02	*PKD2*	Within
	Hapmap54188-rs29022489	6	75017253	1.11E-02	NA	NA
	Hapmap24324-BTC-062449^a^	6	37631640	1.07E-02	*PIGY*	45459
	ARS-BFGL-NGS-111443	5	94269370	1.04E-02	*DERA*	46923
	ARS-BFGL-NGS-107037	10	46486647	9.55E-03	*USP3*	Within
	ARS-BFGL-NGS-61452	4	75250982	9.15E-03	*HUS1*	91623
	ARS-BFGL-NGS-53343	6	29709875	8.08E-03	NA	NA
	ARS-BFGL-NGS-117896	28	35874524	7.50E-03	*MAT1A*	Within
	Hapmap42216-BTA-45665	19	45934555	7.45E-03	*GOSR2*	Within
	Hapmap50621-BTA-21320	6	64425164	7.31E-03	NA	NA
	ARS-BFGL-NGS-53398	X	21953655	7.30E-03	*MAGEA11*	Within
	Hapmap38455-BTA-100999	9	76346736	6.94E-03	*OLIG3*	63196
	BTA-48480-no-rs	2	95119968	6.70E-03	*ADAM23*	25151

a SNP are also detected by SMMA; NA: there is no assigned gene around the SNP in a distance of 200 kb; —: the SNPs with unknown positions.

## Discussion

In this research, a new EM algorithm was developed to simultaneously estimate multiple-SNP effects, which uses an improved lasso prior for marker effects. Usually, the priors of coefficient is very important in multiple linear regression, which may bring special results for shrinking coefficient effects [12]. Most of methods assign the double-exponential prior and Jeffery prior to regression coefficient. We have proposed an improved double-exponential prior for shrinkage estimation of regression coefficients in our previous work [9] that assigns an independent double-exponential prior to model effect and further assigns prior Gamma(0,0) (the BIDE method) or Gamma(0.5,0) (the EMAIL method) to hyperparameter 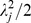. BIDE is implemented via Bayesian MCMC, whereas EMAIL is via EM algorithm. Although MEML is also implemented via EM algorithm, it is different from EMAIL, since (1) it assigns a Gamma prior Gamma(*a*, *b*) with *a* and *b* being very small numbers to the hyper-parameter 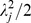; (2) it treats marker variance as missing data and estimates other parameters by searching their maximum posterior mode, while EMAIL adopts Xu's idea who treats the effects of regression coefficient as missing data [13]. The prior Gamma(*a,b*) with *a* and *b* being small numbers are the key of MEML. Although the prior Gamma(0,0) performed well in BIDE, it will not be meaningful in MEML, which can be seen from Equation (12). We also varied *a* and *b* to some other small numbers (smaller than 10^−3^), but no obvious differences were shown.

Yi and Banerjee proposed a hierarchical generalized linear model for multiple QTL mapping [14]. They assigned the variance of model effect 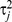 an independent Jeffery prior 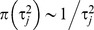, and estimated model effects by finding the maximum posterior mode. It involves the approximation of the generalized likelihood with the weighted normal likelihood in order to analyze binary trait. We modified their method by directly using normal likelihood for the continuous trait and applied their method to our simulated and real dataset. It was found that the results were almost the same as those in MEML (results not shown), which further confirmed the efficiency of MEML.

Following [14], we selected a subset of SNPs with the lowest *p*-values in single-marker analysis for the multiple-SNP analysis. Compared with full-marker analysis (estimate all markers simultaneously), this strategy can save a lot of computational time, so one can use permutation test to ascertain the threshold value to declare the significance of a SNP. In both simulation study and real-data analysis, we selected 500 SNP markers for the analysis, and it performed well. We also varied the selected SNP numbers from 200 to 2000, and no clear differences were found (data not shown), which reflects MEML was not very sensitive to the selected SNP numbers.

In our results from the real data analysis, the SNP ARS-BFGL-NGS-4939 which is located within *DGAT1* on chromosome 14 had the largest effects for all milk production traits. It has been concluded by many researches that a causative mutation in *DGAT1* affects variation in milk production traits. On chromosome 20, a mutation in *GHR* (growth hormone receptor) has been identified to have a large effect on milk yield and composition. The SNP ARS-BFGL-NGS-118998 which is exactly located in this gene was detected to have large effect on fat percentage in our results, but we did not detect this SNP for milk yield. Moreover, Cohen-Zindel et al. identified mutations in the *ABCG2* (ATP-binding cassette sub-family G member 2) gene on chromosome 6 which have an effect on protein percentage [15]. In our results, two SNPs, Hapmap24324-BTC-062449 and BTA-121739-no-rs, were detected for protein percentage and they are very close to the *ABCG2* gene in a distance less than 100 kb. Compared with our previous research by SMMA [7], these functional genes above were all successfully confirmed again by our new method with more targeted mapping, which again validated the new method.

Besides those genes which already investigated in previous studies, in our result, we also detected other potential functional candidate genes nearby these associated SNPs. SNP ARS-BFGL-NGS-11319 associated with milk yield, is located nearby the *SLC40A1* (solute carrier family 40 member 1) gene on chromosome 2. *SLC40A1* encodes ferroportin which is an iron transporter involved in iron export from cell, and it is essential for iron homeostasis. Moreover, Duan et al. found that the polymorphisms of this gene as well as TRF2 (transferrin receptor 2) were significantly associated with beef iron concentration [16]. It is possible that the polymorphisms of SLC40A1 have an effect on iron homeostasis of bovine breast cells, which leads to a potential relationship with milk yield. SNP ARS-BFGL-NGS-39539 located on chromosome 23 and associated with protein yield is close to gene *JARID2* (jumonji, AT rich interactive domain 2) that encodes protein Jumonji (JMJ). JMJ also interacts physically with a transcriptional activator, ZNF496 (zinc finger protein 496), which inhibits the transcriptional repression of JMJ, which plays important roles in embryonic development. Golik et al found that a SNP in bovine *ZNF496* gene displayed significant population-wide linkage disequilibrium with milk protein percentage in the Israeli Holstein population [17], so the interaction between ZNF496 and JMJ as well as our results indicate that *JARID2* also has value to be investigated in further research. SNP ARS-BFGL-NGS-111443 associated with both fat percentage and protein percentage is located between the *DERA* (deoxyribose-phosphate aldolase) gene and the *SLC15A5* (solute carrier family 15, member 5) gene. *SLC15A5* is also a member of the solute carrier family like *SLC40A1* mentioned above, thus it of value to investigated whether it is a causal gene for milk composition.

We have presented the MEML method for multiple-SNP analysis, and it was found to be efficient for genome-wide association study both in simulation experiments and real-data analysis. The key feature of it is that it can dramatically reduce the false-positive SNPs number. Thus, in practice, one can first roughly select many suspicious markers using SMMA and then further confirm them using MEML.

## Materials and Methods

### Multiple-SNP mixed linear model

Consider *n* individuals with *p* SNP markers being investigated, the multiple-SNP model can be expressed as
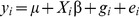
(1)where *y_i_* is the phenotype of the *i*th individual; 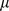 is the population mean; 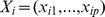 where 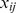 is the genotype of the *j*th SNP marker of the *i*th individual, which is assigned 1, 0 and −1 for genotype *AA*, *Aa* and *aa*, respectively. 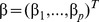, where 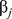 is the additive effect of the *j*th SNP marker; *g_i_* is the residual polygenic effect for the *i*th individual, and *e_i_* is the residual error, which follows a normal distribution, 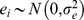.

### Prior specifications

The population mean follows a uniform prior, 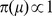. In Baysian Lasso, the regression coefficient 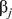 is assigned a double-exponential prior [12],
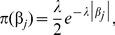
(2)where 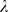 is the hyperparameter. We have modified the Lasso prior by assigning an independent double-exponential prior to each marker effect [9], *i.e.*,
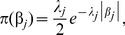
(3)which can be factorized into two-level priors: at the first level, 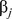 follows a normal distribution,
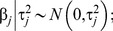
(4)and at the second level, 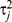 follows an exponential distribution,
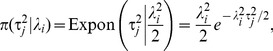
(5)where 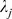 is the hyperparameter and 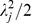 is assigned a conjugate Gamma prior, Gamma(*a*,*b*) with *a* and *b* being very small numbers, and here both *a* and *b* are taken as 10^−6^. The special prior was to have special characters in that it could estimate zero-effect marker effect very close to zero [9]. The prior of the residual variance 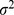 follows non-informative scale-invariant prior 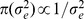; the prior of the residual polygenic effect follows normal distribution 
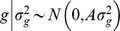
, where 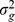 is the residual polygenic variance and *A* is the additive genetic relationship matrix, which can be inferred from pedigree.

### EM algorithm

Let 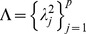, 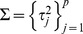, and 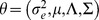; then the likelihood can be expressed as
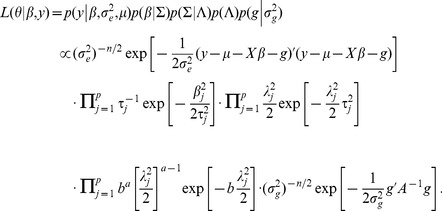
(6)


#### E-step

Since the polygenic effect *g* cannot be derived explicitly, it is treated as missing data here and substituted with its posterior expectation
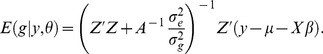
(7)


The posterior variance of *g* is 
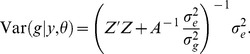
(8)


#### M-step

The M-step maximizes the logarithm of the likelihood (6) 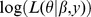 with respect to 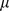, 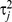, 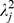, 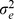 and 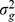 to obtain the their next estimates,
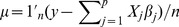
(9)

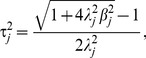
(10)

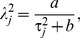
(11)

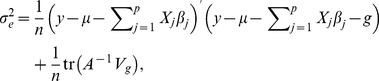
(12)and
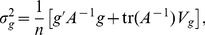
(13)where 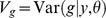.

Given the initial values for 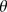, the EM algorithm proceeds with repeatedly updating the E-step equations (7) and (8) and the M-step equations (9)–(13) until reaching convergence.

### Significance test

The variance of the *j*th SNP can be expressed as 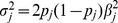, where *p_j_* is the allele frequency of the *j*th SNP. The heritability of the SNP can be written as 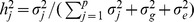. The threshold value for declaring the significance of the SNP is determined from the empirical distribution of 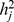 derived by 1,000 permutations.
